# Semaglutide Plus Low-Dose Metformin Combination Therapy for the Treatment of Obesity and Prediabetes in a Woman with Partial Deletion of the X Chromosome Long Arm

**DOI:** 10.3390/reports9010075

**Published:** 2026-02-28

**Authors:** Vincenzo Marzolla, Stefania Gorini, Massimiliano Caprio, Marco Infante

**Affiliations:** 1 Laboratory of Cardiovascular Endocrinology, IRCCS San Raffaele Roma, Via di Val Cannuta 247, 00166 Rome, Italy; nutrizionemarzolla@gmail.com (V.M.); stefania.gorini@uniroma5.it (S.G.); massimiliano.caprio@sanraffaele.it (M.C.); 2 Department for the Promotion of Human Sciences and Quality of Life, San Raffaele Open University, Via di Val Cannuta 247, 00166 Rome, Italy; 3 Section of Diabetes & Metabolic Disorders, Faculty of Medicine and Surgery, UniCamillus—Saint Camillus International University of Health Sciences, Via di Sant’Alessandro 8, 00131 Rome, Italy; 4 Cell Transplant Center, Diabetes Research Institute (DRI), Division of Cellular Transplantation, Department of Surgery, University of Miami Miller School of Medicine, 1450 NW 10th Ave., Miami, FL 33136, USA; 5 Network of Immunity in Infection, Malignancy and Autoimmunity (NIIMA), Universal Scientific Education and Research Network (USERN), Endocrinology, Diabetes and Obesity Outpatient Clinic, Via Cola di Rienzo 28, 00192 Rome, Italy

**Keywords:** X chromosome long arm deletion, partial Xq deletion, obesity, insulin resistance, prediabetes, body composition, incretin analogs, semaglutide, GLP-1 receptor agonists, metformin, case report

## Abstract

**Background and Clinical Significance**: Over the last two decades, glucagon-like peptide-1 (GLP-1) receptor agonists have dramatically improved the management of type 2 diabetes mellitus and obesity. Currently, little is known about the use of semaglutide (a second-generation GLP-1 receptor agonist) in patients with X chromosome abnormalities. Herein, we describe the therapeutic use of semaglutide in a woman with a partial deletion of the X chromosome long arm (partial Xq deletion) and comorbid obesity. We also conducted a narrative mini-review on overweight, obesity and common metabolic derangements in patients with partial Xq deletions and Turner syndrome. **Case Presentation**: A 65-year-old Italian woman with a partial Xq deletion, class 1 obesity, insulin resistance, prediabetes, hypercholesterolemia and metabolic dysfunction-associated steatotic liver disease (MASLD) was referred to our Institution for persistent difficulty in managing excess body weight despite regular adherence to different structured physical activity programs and hypocaloric diets. Therefore, we prescribed a combination therapy based on low-dose metformin (500 mg/day) and once-weekly subcutaneous semaglutide (as an adjunct to lifestyle intervention). At 5 months after initiation of the combination therapy, blood tests showed metabolic improvements, including improvement of prediabetes (0.3-percentage-point reduction in glycated hemoglobin [HbA1c] values) and normalization of markers of insulin sensitivity and insulin resistance (QUICKI, HOMA-IR and TyG index). At 8 months, the patient showed substantial weight loss, which amounted to 13.8 kg (percent total body weight loss: 20.95%), and was accompanied by a notable reduction in waist circumference (−14.1 cm). Moreover, body mass index (BMI)-based weight status improved from class 1 obesity to overweight: BMI value of 25.1 kg/m^2^ at 8 months vs. 31.8 kg/m^2^ at baseline (near-normalization of BMI values). Bioelectrical impedance analysis (BIA) revealed that the patient’s overall weight loss consisted of 74.6% fat mass (FM) loss (−10.3 kg) and 25.4% fat-free mass (FFM) loss (−3.5 kg). Despite the expected FFM reduction in absolute terms, percent FFM increased at 8 months (+9.6%). This increase in percent FFM was accompanied by a reduction in percent FM at 8 months (−9.6%), indicating an overall improvement in body composition. Normalization of percent FM and FFM values (28.6% and 71.4%, respectively) was also achieved at 8 months. These body composition changes are in line with those observed in clinical trials investigating the use of semaglutide in patients with overweight or obesity. At 6 months, an abdominal ultrasound also showed the disappearance of the sonographic characteristics suggestive of mild-to-moderate hepatic steatosis. Low-dose metformin (500 mg/day) and subcutaneous semaglutide (up to a weekly dose of 1.7 mg) were well tolerated by the patient. **Conclusions**: To the best of our knowledge, this is the first case documenting the effective use of once-weekly subcutaneous semaglutide plus low-dose metformin combination therapy for the treatment of obesity and prediabetes in a woman with a partial Xq deletion. Large prospective cohort studies are warranted to better investigate the safety and efficacy profile of semaglutide (alone or in combination with metformin) in patients with numerical and structural X chromosome abnormalities, comorbid overweight/obesity and related metabolic disorders.

## 1. Introduction and Clinical Significance

Incretin analogs are drugs that mimic the physiologic actions of gut-derived hormones (a.k.a. incretins or incretin hormones) secreted from enteroendocrine cells in response to food ingestion [1,2]. Glucagon-like peptide-1 (GLP-1) is an incretin hormone secreted from enteroendocrine L cells (located in the distal gut) in response to food ingestion and is rapidly degraded by the ubiquitous enzyme called dipeptidyl peptidase-4 (DPP-4) [1,2,3]. Over the last two decades, incretin analogs have dramatically improved the management of type 2 diabetes mellitus (T2DM) and obesity, two major chronic conditions that have now reached pandemic proportions [4,5,6]. Semaglutide is a second-generation incretin analog that acts as a GLP-1 receptor agonist (GLP-1 RA) [7]. As a GLP-1 RA, semaglutide potentiates glucose-dependent insulin secretion from pancreatic beta cells, suppresses glucagon secretion from pancreatic alpha cells, slows gastric emptying, promotes satiety and reduces food intake by acting in regions of the central nervous system involved in the regulation of appetite [8].

Semaglutide is approved for the treatment of T2DM and for chronic weight management in patients with obesity or overweight associated with at least one weight-related comorbidity (e.g., hypertension, dyslipidemia, obstructive sleep apnea, prediabetes or T2DM) [7,9]. Moreover, semaglutide is approved for the treatment of obesity in pediatric patients aged 12 years and older [7]. Recently, semaglutide has also been approved for additional indications based on the results of major clinical trials, namely: (i) to reduce cardiovascular risk in adults with overweight/obesity and/or T2DM [10,11]; (ii) to reduce the risk of worsening kidney disease, kidney failure, and death related to cardiovascular disease in adults with T2DM and chronic kidney disease (CKD) [12]; and (iii) to treat metabolic dysfunction-associated steatohepatitis (MASH) in adults [13].

On the other hand, metformin is an extensively used glucose-lowering medication that acts by decreasing hepatic glucose production, enhancing insulin sensitivity, reducing small intestinal glucose absorption, increasing endogenous GLP-1 secretion, modifying gut microbiome and increasing glucose utilization by the gut [14,15,16]. Although metformin is most commonly used for the treatment of T2DM, it can also be prescribed to prevent or delay the progression of prediabetes to T2DM [17].

Structural X chromosome abnormalities (including deletions, duplications, inversions and translocations) have been associated with obesity [18,19,20,21,22,23,24,25,26]. Currently, little is known about the use of semaglutide in patients with X chromosome abnormalities and comorbid overweight/obesity and metabolic disorders. A better understanding of the safety and efficacy profile of semaglutide in these patients is clinically relevant, given the global rise in the prevalence of T2DM and overweight/obesity [4,6], a trend that has recently paralleled a widespread increase in the prescription of GLP-1 receptor agonists (GLP-1 RAs) [27,28,29]. Herein, we describe the case of a 65-year-old woman with a partial deletion of the X chromosome long arm, class 1 obesity, prediabetes, hypercholesterolemia and metabolic dysfunction-associated steatotic liver disease (MASLD), who experienced substantial weight loss accompanied by body composition and metabolic improvements after initiating combination therapy with once-weekly subcutaneous semaglutide and low-dose metformin (in addition to lifestyle intervention). Moreover, we conducted a narrative mini-review on overweight, obesity and common metabolic derangements in patients with partial Xq deletions and Turner syndrome.

## 2. Case Presentation

**Figure 1:**
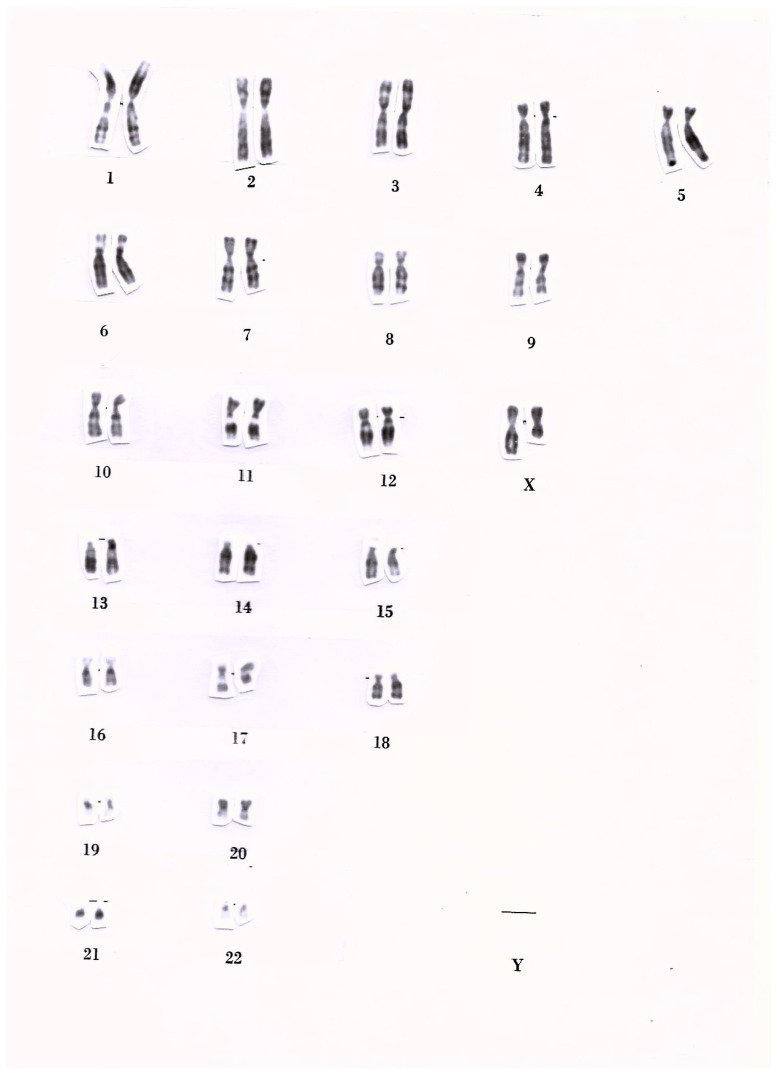
Karyotype of peripheral blood lymphocytes from the patient. Karyotype analysis was performed when the patient was 20 years old and revealed a partial deletion of the X chromosome long arm (partial Xq deletion), as follows: 46,XX,del(Xq)(Xpter → q21:). Chromosome banding was performed using the G-banding method (with Giemsa stain). Horizontal black lines appearing beneath some chromosome photographs are alignment markers that were previously used to position printed photographs of metaphase chromosomes (homologous chromosome pairs and the pair of sex chromosomes) on the karyotype template sheet so that the centromeres were aligned at the same height.

A 65-year-old Italian woman was referred to our Endocrinology, Diabetes and Obesity Outpatient Clinic for obesity. The patient reported persistent difficulty in managing excess body weight since approximately 37 years of age, despite regular adherence to different structured physical activity programs and low- and very-low-calorie diets (including very-low-calorie ketogenic diets) prescribed and monitored under dietitian supervision. Based on the patient’s weight history, previous weight loss with the aforementioned lifestyle interventions was less than 5% of her initial body weight. The patient was nulligravid and had short stature associated with a medical history of premature menopause. She was born at term following an uncomplicated vaginal delivery. She reported menarche at 9 years of age. After menarche, she experienced persistent menstrual irregularities that ultimately resulted in secondary amenorrhea. At 20 years of age, she developed primary ovarian insufficiency, resulting in premature menopause. Then, she underwent karyotype analysis and she was diagnosed with a partial deletion of the X chromosome long arm (partial Xq deletion). The patient’s karyotype was 46,XX,del(Xq)(Xpter → q21:). Figure 1 shows the karyotype of peripheral blood lymphocytes from our patient. The patient subsequently received oral postmenopausal hormone replacement therapy until 50 years of age. 

The patient reported a family history of T2DM (father and maternal grandfather) and breast cancer (mother). Moreover, the patient suffered from other conditions, namely: prediabetes, hypercholesterolemia, MASLD, gastroesophageal reflux disease (GERD), subcentimeter thyroid nodules, and lumbar degenerative disk disease. The patient also reported having undergone an appendectomy for appendicitis and an adenoidectomy for adenoid hypertrophy (both surgical procedures were performed when the patient was 7 years old). She was periodically monitored by a cardiologist, although she had no history of cardiovascular disease, congenital heart defects, or prior cardiovascular events. She reported no known allergies or intolerances, including food and medication allergies or intolerances. At the time of clinical presentation, the patient reported physical inactivity over the previous 3 months due to reduced motivation following unsuccessful weight loss attempts, despite previous regular adherence to structured physical activity programs and hypocaloric diets. She denied significant alcohol consumption and any history of smoking or illicit drug use. She was taking omeprazole (20 mg capsules, 1 capsule/day, taken orally in the morning on an empty stomach) and ezetimibe/simvastatin (10 mg/20 mg combination tablets, 1 tablet/day, taken orally prior to bedtime) for the treatment of GERD and hypercholesterolemia, respectively. Body height and weight were assessed using a digital scale with an integrated height-measuring rod (digital scale no. 7831; Soehnle Industrial Solutions GmbH, Backnang, Germany). Body circumferences were assessed using three-dimensional (3D) body scanning technology—as previously described [30]—with the Fit3D ProScanner (Fit3D ProScanner v5.0; Fit3D, Inc.; San Mateo, CA, USA), a device based on infrared technology that has been validated for high precision [30,31]. Body composition assessment was carried out at the IRCCS San Raffaele Clinical Trial Center (Rome, Italy) by bioelectrical impedance analysis (BIA), using the BIA 101 BIVA^®^ PRO (Akern S.r.l., Pontassieve, Florence, Italy). Skeletal muscle mass (SMM) was estimated using the Janssen’s equation, as previously described [32]. Both 3D body scanning and BIA were performed after ensuring that the patient had fasted for 10 h, refrained from exercise and alcohol consumption for the preceding 24 h, and emptied her bladder immediately prior to 3D body scanning and BIA measurements. Before undergoing BIA, the patient rested quietly in a supine position at a stable ambient temperature (approximately 24 °C) for at least 5 min.

At the time of referral to our Outpatient Clinic (May 2025), the patient’s height and weight were 144 cm and 65.9 kg, respectively, with a body mass index (BMI) value indicative of class 1 obesity (31.8 kg/m^2^) [33]. Values of waist circumference (WC: 101.2 cm), waist-to-hip ratio (WHR) and waist-to-height ratio (WHtR) were indicative of central (visceral) obesity and increased cardiometabolic risk (0.95 and 0.70, respectively; Table 1) [34,35]. Blood pressure values were 120/80 mmHg, heart rate was 82 bpm (regular arterial pulse rhythm), and oxygen saturation (SpO_2_) was 97%.

Thyroid Doppler ultrasound showed a thyroid gland of normal volume with smooth and well-defined margins, as well as normal sonographic appearance and orientation of the trachea. The thyroid parenchyma showed slightly inhomogeneous echogenicity, with evidence of two subcentimeter thyroid nodules (one in the right lobe and one in the left lobe): both nodules were solid and hypoechoic, with an oval shape, smooth margins, and peripheral vascularity. No alterations in thyroid parenchymal vascularity were observed. There was no ultrasonographic evidence of abnormal cervical lymph nodes.

Blood tests (performed after a 10-h overnight fast) showed a glycated hemoglobin (HbA1c) value indicative of prediabetes (6.0%; 42.0 mmol/mol) [36] [in the presence of normal fasting plasma glucose (FPG) levels: 96 mg/dL], homeostatic model assessment for insulin resistance (HOMA-IR), quantitative insulin sensitivity check index (QUICKI), and triglyceride-glucose (TyG) index values (calculated as previously described) [37,38,39] indicative of insulin resistance (3.5, 0.317 and 4.77, respectively) [37,38,39], hypovitaminosis D (serum 25-hydroxyvitamin D: 12.14 ng/mL) [40], mildly elevated gamma–glutamyl transferase (GGT) (39 U/L; reference range: 2.0–35.0 U/L), and slightly elevated creatine phosphokinase (CPK) and alpha-1 globulin values (189.0 U/L and 4.6 g/L, respectively), as well as low-density lipoprotein (LDL) cholesterol and non-high-density lipoprotein (non-HDL) cholesterol values above target levels (102.2 mg/dL and 131 mg/dL, respectively) [Supplementary Table S1]. Results of other laboratory tests did not reveal remarkable findings (Supplementary Table S1). Values of markers of thyroid function, calcitonin, anti-thyroid peroxidase antibodies and anti-thyroglobulin antibodies were within the reference range (Supplementary Table S1). At baseline, LDL cholesterol was calculated using the Friedewald equation [41]. The estimated glomerular filtration rate (eGFR) was calculated using the 2021 Chronic Kidney Disease Epidemiology Collaboration (CKD-EPI) equation [42]. Notably, blood tests performed 12 months earlier had already shown HbA1c values indicative of prediabetes (5.9%; 41.0 mmol/mol) [36], as well as a HOMA-IR value of 3.0 [fasting plasma glucose: 99 mg/dL; fasting insulinemia: 12.14 μIU/mL], indicative of insulin resistance [37]. Therefore, we increased the simvastatin dose, as follows: ezetimibe/simvastatin 10 mg/40 mg combination tablets, 1 tablet/day, taken orally prior to bedtime (in place of ezetimibe/simvastatin 10 mg/20 mg combination tablets, 1 tablet/day). Moreover, we prescribed vitamin D3 (cholecalciferol) supplementation to correct hypovitaminosis D, as follows: vitamin D3 4000 IU tablets, 1 tablet/day (taken orally at breakfast).

### 2.1. Semaglutide Plus Low-Dose Metformin Combination Therapy: Initiation, Titration and Management

Given the patient’s clinical history (including weight history) and laboratory test results, we prescribed semaglutide plus low-dose metformin combination therapy as an adjunct to lifestyle interventions (hypocaloric diet and regular physical activity) for the management of obesity and prediabetes. Once-weekly subcutaneous semaglutide (Wegovy^®^; Novo Nordisk, Bagsværd, Denmark) was prescribed at a dose of 0.25 mg/week, which is the standard starting drug dose for semaglutide-naïve patients [7]. In view of the laboratory test results indicative of prediabetes and insulin resistance, the patient was also prescribed a low daily dose of metformin (Slowmet^®^; Savio Pharma Italia S.r.l., Pomezia, Italy—500 mg metformin extended-release tablets, 1 tablet/day, taken shortly after the evening meal). Metformin therapy was initiated to manage prediabetes and prevent its progression to T2DM, in accordance with international guidelines [17]. A low dose of extended-release metformin was chosen to minimize potential overlap and/or synergism between gastrointestinal adverse effects shared by metformin and GLP-1 RAs (particularly nausea, vomiting, diarrhea and constipation) [43,44,45,46].

With regard to lifestyle interventions, we prescribed a hypocaloric, low-carbohydrate dietary regimen characterized by a daily protein intake of approximately 1.3 g/kg of actual body weight. We also recommended a structured program of mild-intensity aerobic physical activity combined with strength and resistance training exercises (approximately 180 min of physical activity per week, spread over three days; about 60 min per exercise session). We also advised the patient to aim for approximately 7000 steps per day. The aforementioned dietary and physical activity recommendations aligned with recent evidence-based strategies aimed at preserving lean mass loss and promoting healthy weight loss in patients with overweight/obesity treated with weight-loss drugs [47]. Moreover, we recommended a minimum daily water intake of 2 L, which corresponds to approximately 30 mL of water/kg of body weight per day.

Patient adherence to lifestyle interventions (hypocaloric diet and regular physical activity) and pharmacotherapy was regularly evaluated through clinical nutrition and obesity follow-up outpatient consultations, follow-up laboratory tests and periodic assessment of body composition and body circumferences.

After 4 weeks, the weekly semaglutide dose was gradually up-titrated over the subsequent months, according to the standard drug dose escalation schedule [7,9], as follows: second month of semaglutide therapy, 0.5 mg/week; third month of semaglutide therapy, 1 mg/week; fourth month of semaglutide therapy, 1.7 mg/week; and fifth month of semaglutide therapy, 2.4 mg/week. However, at 6 months after initiation of semaglutide therapy, the weekly semaglutide dose was decreased to 1.7 mg/week, since the patient started to experience progressive generalized dysesthesia during the use of the highest weekly semaglutide dose (2.4 mg/week). Specifically, the patient reported a mild-to-moderate sunburn-like skin sensation. Yet, dysesthesia resolved rapidly after the weekly semaglutide dose was reduced to 1.7 mg. The weekly semaglutide dose of 1.7 mg was well tolerated by the patient. However, after the subsequent 4 weeks of semaglutide therapy at a dose of 1.7 mg/week, we decided to reduce the weekly drug dose to 1 mg in order to maintain fat mass (FM) loss and mitigate the decline in fat-free mass (FFM) observed on follow-up BIA (Table 2). The dosing regimen used for semaglutide therapy during the follow-up period is shown in Table 3.

During the entire 8-month follow-up period, low-dose metformin (500 mg/day) and subcutaneous semaglutide (up to a weekly dose of 1.7 mg) were well tolerated by the patient. During this period, the patient regularly monitored capillary blood glucose and blood pressure values at home using a glucometer and an automatic digital blood pressure monitor. There were no relevant gastrointestinal side effects, hypoglycemic episodes, hypotensive events or other adverse drug reactions (other than transient dysesthesia) during the entire 8-month follow-up period.

At 5 months after initiation of semaglutide plus low-dose metformin combination therapy, the patient showed a 0.3-percentage-point reduction in HbA1c values (5.7% vs. 6.0% at baseline), normalization of HOMA-IR, QUICKI and TyG index values (1.6, 0.355, and 4.49, respectively) [37,38,39], as well as normalization of serum GGT, CPK and alpha-1 globulin values (Supplementary Table S1). Moreover, serum 25-hydroxyvitamin D [25(OH)D] values became sufficient (39.0 ng/mL) [40] at 5 months following the initiation of vitamin D3 supplementation, in the presence of normal total serum calcium values (9.50 mg/dL). At 5 months after initiation of semaglutide plus low-dose metformin combination therapy and the lipid-lowering therapy intensification, serum LDL cholesterol and non-HDL cholesterol values decreased to desired target levels (50 mg/dL and 75 mg/dL, respectively; Supplementary Table S1) [48,49]. Serum folate (vitamin B9), vitamin B12 and homocysteine values were within the reference range at 5 months after initiation of semaglutide plus low-dose metformin combination therapy (Supplementary Table S1). The latter results largely excluded folate deficiency or vitamin B12 deficiency as potential contributors to the development of dysesthesia, which was reported by the patient 5 months after initiation of semaglutide plus low-dose metformin combination therapy. Although serum methylmalonic acid (MMA) was not measured, true vitamin B12 deficiency was unlikely, since serum vitamin B12 levels were higher than the borderline-low values reported in other studies (>298 pg/mL) and serum homocysteine values were normal (9.0 μmol/L) [50,51,52]. Results of other laboratory tests (including serum amylase and lipase) did not reveal remarkable findings (Supplementary Table S1).

An electrocardiogram (ECG) performed at 6 months after initiation of semaglutide plus low-dose metformin combination therapy did not reveal abnormal findings. The ECG showed a heart rate of 78 bpm, in the presence of normal sinus rhythm, normal intraventricular and atrioventricular conduction, normal ventricular repolarization, and normal cardiac axis. Compared with an abdominal ultrasound performed 4 years earlier, the abdominal ultrasound performed at 6 months after initiation of semaglutide plus low-dose metformin combination therapy showed the disappearance of the sonographic characteristics suggestive of mild-to-moderate hepatic steatosis (bright liver echo pattern and increased echogenicity of the liver compared to the kidney and spleen echogenicity) [53,54] and the presence of normal liver size. The ovaries and fallopian tubes could not be visualized, while there was sonographic evidence of uterine atrophy (in line with the patient’s age and clinical history). Moreover, a follow-up thyroid Doppler ultrasound (performed at 6 months after initiation of semaglutide plus low-dose metformin combination therapy) did not reveal changes in the size and sonographic characteristics of thyroid nodules compared with the baseline thyroid Doppler ultrasound performed 6 months earlier.

At 8 months after initiation of semaglutide plus low-dose metformin combination therapy, the patient reported an improvement in exercise capacity and overall well-being compared with the pre-treatment period. At the end of the 8-month follow-up period, blood pressure values were 120/70 mmHg and SpO_2_ was 99%.

### 2.2. Changes in Anthropometric Parameters, Body Circumferences and BIA Parameters After Initiation of Semaglutide Plus Low-Dose Metformin Combination Therapy

At the end of the follow-up period (at 8 months after initiation of semaglutide plus low-dose metformin combination therapy), the patient experienced substantial weight loss, which amounted to 13.8 kg (corresponding to a percent total body weight loss [%TBWL] of 20.95%). At 8 months after initiation of semaglutide plus low-dose metformin combination therapy, this weight loss was associated with the attainment of a BMI value indicative of overweight, compared with a baseline BMI value indicative of class 1 obesity: 25.1 kg/m^2^ at 8 months vs. 31.8 kg/m^2^ at baseline (−6.7 kg/m^2^) [33]. Moreover, this weight loss was accompanied by a 14.1-cm reduction in WC, a 0.04 reduction in WHR and a 0.10 reduction in WHtR. Importantly, the patient’s weight loss consisted of a 10.3-kg reduction in FM, which resulted in a 9.6-percentage-point decrease in percent fat mass (%FM). Although there was a 3.5-kg reduction in FFM, the percent fat-free mass (%FFM), percent skeletal muscle mass (%SMM), and percent appendicular skeletal muscle mass (%ASMM) increased (+9.6%, +5.2%, and +2.7%, respectively) at the end of the 8-month follow-up period (Table 2). The patient’s overall weight loss (−13.8 kg) consisted of 74.6% FM loss (−10.3 kg) and 25.4% FFM loss (−3.5 kg). There were also reductions in the following BIA parameters: FM/FFM ratio (−0.21); body cell mass (BCM; −0.7 kg), although this was associated with a 3.6-percentage-point increase in percent BCM; phase angle (PhA; −0.4°); total body water (TBW; −3.7 L); extracellular water (ECW; −2.2 L); intracellular water (ICW; −1.5 L); ECW/TBW ratio (−0.014) (Table 2). Changes in other BIA parameters are shown in Table 2.

Surprisingly, at 8 months after initiation of semaglutide plus low-dose metformin combination therapy, while the patient was receiving the weight maintenance dose of semaglutide (1 mg/week), she exhibited an additional FM loss (−2.1 kg) compared with the FM value observed at 6 months (during treatment with a weekly semaglutide dose of 1.7 mg) (Table 2). Remarkably, this additional FM loss was accompanied by increases in FFM (+0.8 kg), SMM (+1.0 kg) and ASMM (+0.3 kg) values compared with the FFM, SMM and ASMM nadir values observed at 6 months (during treatment with a weekly semaglutide dose of 1.7 mg) (Table 2). Overall, body composition changes observed at 8 months with the weight maintenance dose of semaglutide (1 mg/week) resulted in the normalization of percent FM and percent FFM (28.6% and 71.4%, respectively; Table 2) based on established reference values for these BIA parameters [55].

**Figure 2:**
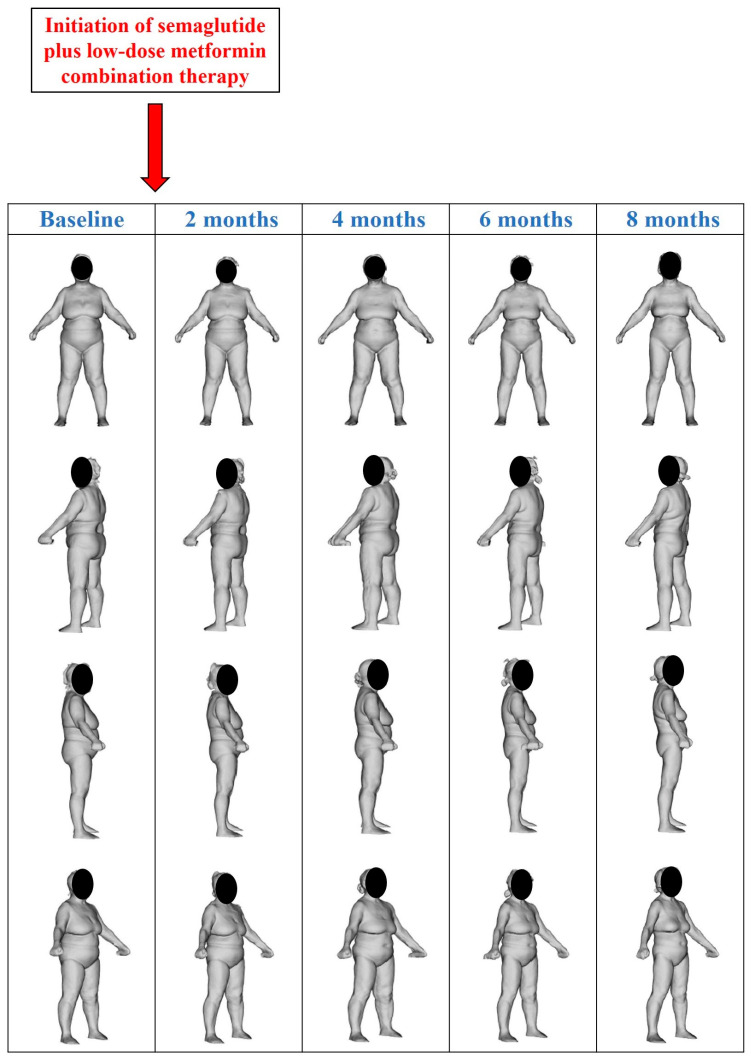
Three-dimensional (3D) body surface images obtained with 3D body scanning technology at baseline (before initiation of semaglutide plus low-dose metformin combination therapy) and during the follow-up period. 3D body scanning was performed using the Fit3D ProScanner (Fit3D ProScanner v5.0; Fit3D, Inc.; San Mateo, CA, USA).

**Figure 3:**
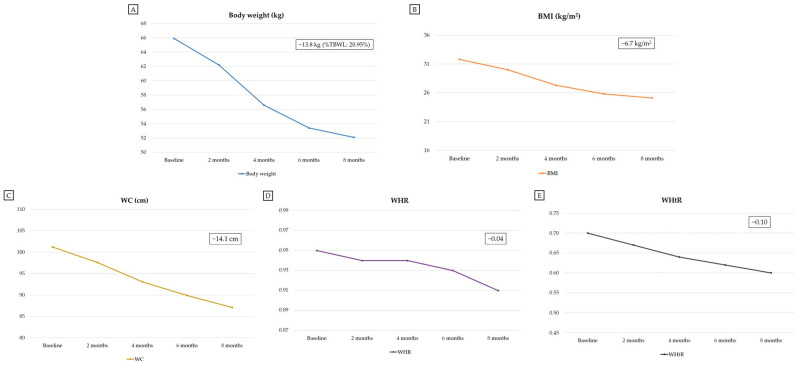
Changes from baseline (before initiation of semaglutide plus low-dose metformin combination therapy) in the main anthropometric parameters during the follow-up period. The figure shows changes in body weight (**A**), BMI (**B**), WC (**C**), WHR (**D**) and WHtR (**E**) during the follow-up period. Abbreviations: %TBWL, percent total body weight loss; BMI, body mass index; WC, waist circumference; WHR, waist-to-hip ratio; WHtR, waist-to-height ratio.

**Figure 4:**
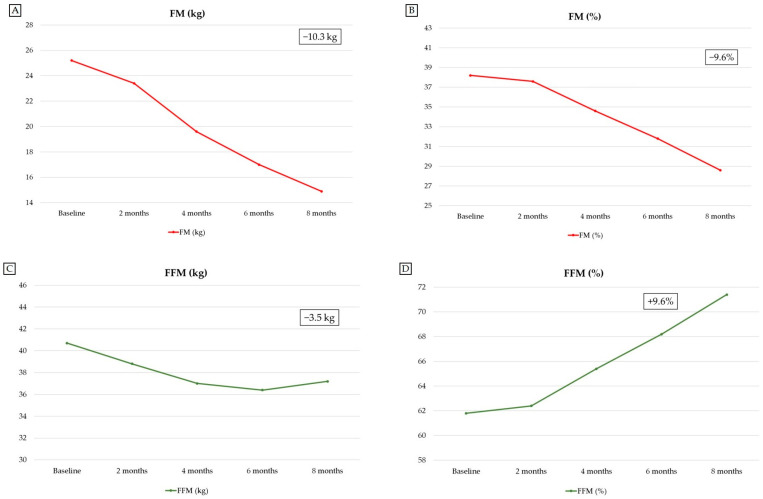
Changes from baseline (before initiation of semaglutide plus low-dose metformin combination therapy) in FM and FFM (assessed by bioelectrical impedance analysis [BIA]) during the follow-up period. FM and FFM are reported as both absolute values (expressed in kg) and percentage (%) values. The figure shows changes in FM (kg) (**A**), FM (%) (**B**), FFM (kg) (**C**) and FFM (%) (**D**) during the follow-up period. Abbreviations: FFM, fat-free mass; FM, fat mass.

**Figure 5:**
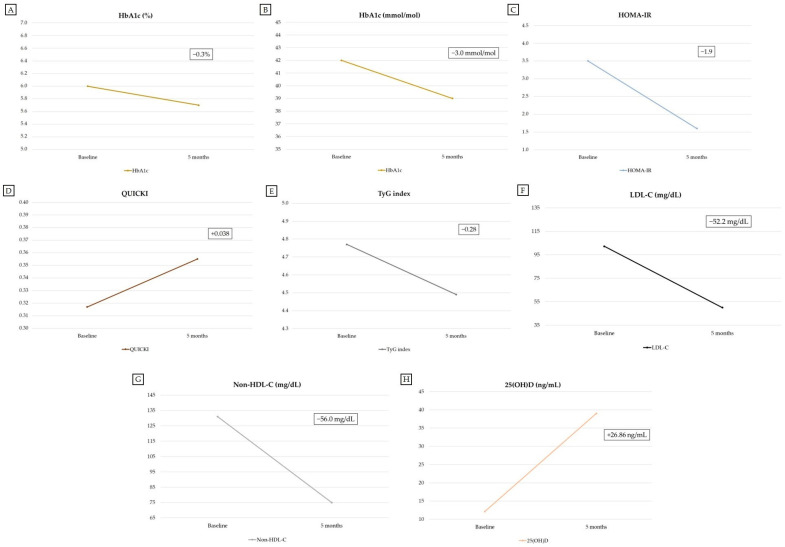
Changes from baseline (before initiation of semaglutide plus low-dose metformin combination therapy and vitamin D3 supplementation) in the main blood parameters during the follow-up period. The figure shows changes in HbA1c (%) (**A**), HbA1c (mmol/mol) (**B**), HOMA-IR (**C**), QUICKI (**D**), TyG index (**E**), LDL-C (**F**), Non-HDL-C (**G**) and 25(OH)D (**H**) during the follow-up period. Abbreviations: 25(OH)D, 25-hydroxyvitamin D; HbA1c, glycated hemoglobin; HOMA-IR, homeostatic model assessment for insulin resistance index; LDL-C, low-density lipoprotein cholesterol; Non-HDL-C, non-high-density lipoprotein cholesterol; QUICKI, quantitative insulin sensitivity check index; TyG index, triglyceride-glucose index.

Data regarding the patient’s anthropometric parameters and body circumferences at baseline (before initiation of semaglutide plus low-dose metformin combination therapy) and during the follow-up period are shown in Table 1. Data regarding the patient’s BIA parameters at baseline (before initiation of semaglutide plus low-dose metformin combination therapy) and during the follow-up period are shown in Table 2. Table 3 shows the dosing regimen used for semaglutide therapy during the follow-up period. Figure 2 shows the 3D body surface images obtained with 3D body scanning technology at baseline (before initiation of semaglutide plus low-dose metformin combination therapy) and during the follow-up period. Changes from baseline in the main anthropometric parameters, BIA parameters (FM and FFM), and blood parameters during the follow-up period are shown in Figure 3, Figure 4 and Figure 5, respectively.

## 3. Discussion

### 3.1. Overweight, Obesity and Common Metabolic Derangements in Patients with Partial Xq Deletions and Turner Syndrome: A Narrative Mini-Review

Structural X chromosome abnormalities have been associated with obesity. These structural X chromosome abnormalities include deletions [18,19], duplications [20,21,22,23], inversions [24,25] and translocations [26]. Dasouki et al. [18] previously reported the case of an 8-year-old Caucasian girl with early-onset truncal obesity and a de novo germline deletion involving the chromosome Xq27.1-q28 region, where a large 10.69 Mb deletion was found [base position: 139,354,859–150,046,723; hg18]. The patient experienced global developmental delay associated with slow postnatal weight gain and exhibited other clinical features, including short stature, microcephaly, myopathic facies, facial dysmorphism, pinched nostrils, small mouth and earlobes, small hands and feet, prominent philtrum, long and narrow face, prominent forehead, narrow palate, prognathism, ptosis, right esotropia, hypotonia, and early feeding difficulties, as well as severe speech and cognitive delays [18].

Primary ovarian insufficiency has already been reported in female patients with partial deletions of the long arm of the X chromosome involving Xq critical regions for normal ovarian function [56,57,58]. Our patient exhibited clinical features overlapping with some of those commonly observed in patients with Turner syndrome, namely short stature, premature menopause, insulin resistance and prediabetes [59,60]. Turner syndrome represents one of the most common sex chromosome abnormalities, resulting from total or partial monosomy of the X chromosome and affecting about 1 in 2000 to 2500 female newborns [61,62]. Moreover, Turner syndrome associated with partial deletions of the X chromosome long arm has been reported in the literature, even though affected subjects generally exhibit only a few clinical features of the syndrome [63,64]. Lippe and Crandall [65] previously described the case of an 18-year-old girl with Turner syndrome associated with partial Xq deletion and characterized by short stature, elevated serum gonadotropin levels and secondary amenorrhea. Secondary amenorrhea has also been described in the presence of partial Xq deletion and in the absence of clinical features of Turner syndrome [66]. Dysglycemia can also be present in patients with Turner syndrome associated with partial deletions of the X chromosome long arm [63]. In general, our patient had short stature, primary ovarian insufficiency (accompanied by premature menopause at 20 years of age), insulin resistance and prediabetes, although she did not exhibit other clear stigmata of Turner syndrome, including skeletal abnormalities, shield chest, lymphedema, webbed neck, congenital heart defects, renal malformations and autoimmune disorders [67,68,69,70].

Overweight, obesity, excess visceral adipose tissue and hepatic fat accumulation are frequently observed in patients with Turner syndrome [71,72,73]. Studies have shown that patients with Turner syndrome, compared with matched controls, exhibit a higher prevalence of obesity (including truncal obesity), metabolic syndrome, prediabetes, dyslipidemia, hypertension, and hepatic steatosis, as well as greater total body fat percentage and fat mass index values and lower lean mass index values [74,75]. Although the exact cause of the increased risk of dysglycemia and diabetes mellitus (DM) observed in patients with Turner syndrome is poorly understood, recent data suggested a potential role of maternal X chromosome monosomy [76]. Turner syndrome-associated DM is accompanied by insulin resistance (evidenced by elevated fasting insulin and HOMA-IR values compared to counterparts without DM), which is not completely accounted for by body weight, since the HOMA-IR can be significantly increased even when controlled for BMI [77]. Thus, it has been hypothesized that insulin resistance may represent an innate feature of Turner syndrome that can be present before the onset of DM, being partly independent of excess body weight and attributable to various factors, such as haploinsufficiency of X-chromosome genes affecting insulin action and/or relative resistance to the insulin-sensitizing effects of estrogen [77]. In this regard, Caprio et al. [78] already documented that insulin resistance is a very early metabolic defect in patients with Turner syndrome. Cameron-Pimblett et al. [77] showed that the median age of onset of diabetes diagnosis in women with Turner syndrome was 36 years, which falls midway between population references for type 1 diabetes mellitus (T1DM) and T2DM. This may suggest Turner syndrome-associated DM as a distinct diabetes entity or a mixed form of T1DM and T2DM, with features of both these types of DM [77]. Indeed, Cameron-Pimblett et al. [77] also found that women with Turner syndrome were 8.5 times more likely to exhibit positivity for glutamic acid decarboxylase autoantibodies (GADA) compared with the general population. Moreover, the presence of both beta-cell dysfunction and reduced insulin sensitivity suggests a unique glycemic phenotype in patients with Turner syndrome and dysglycemia [79].

Given the aforementioned remarks, incretin analogs (including GLP-1 RAs) have been proposed as valid drugs able to effectively treat insulin resistance and prevent the development or improve the management of prediabetes or DM in patients with Turner syndrome [80]. Additionally, semaglutide and other incretin analogs may represent a valid therapeutic strategy for the management of Turner syndrome-associated overweight/obesity and related comorbidities (including prediabetes, MASLD, dyslipidemia and hypertension) [73,75,80] and for the reduction of cardiovascular risk in these patients, who are at higher risk for congenital heart disease, early-onset hypertension, atherosclerotic cardiovascular disease, ischemic heart disease and stroke [81,82].

### 3.2. Clinical Case Discussion 

To the best of our knowledge, this is the first case documenting the effective use of once-weekly subcutaneous semaglutide plus low-dose metformin combination therapy for the treatment of obesity and prediabetes in a woman with a partial Xq deletion.

In our patient, partial Xq deletion did not appear to negatively affect the therapeutic response to semaglutide plus low-dose metformin combination therapy. Indeed, the 8-month semaglutide plus low-dose metformin combination therapy led to substantial weight loss (−13.8 kg; %TBWL: 20.95%), with marked improvement in BMI-based weight status and near-normalization of BMI values (from class 1 obesity to overweight; BMI of 25.1 kg/m^2^ at 8 months vs. 31.8 kg/m^2^ at baseline) [33]. Moreover, the 8-month semaglutide plus low-dose metformin combination therapy led to reductions in the values of anthropometric parameters indicative of visceral obesity (WC: −14.1 cm; WHR: −0.04; WHtR: −0.10) [83,84,85]. The patient’s overall weight loss (−13.8 kg) consisted of 74.6% FM loss (−10.3 kg) and 25.4% FFM loss (−3.5 kg). Thus, the patient’s weight loss was accompanied by body composition improvement, as evidenced by predominant FM loss (−10.3 kg), reduction of percent FM (−9.6%), increase of percent FFM (+9.6%), percent SMM (+5.2%) and ASMM (+2.7%), and reduction of the FM/FFM ratio (−0.21). Surprisingly, the normalization of percent FM and FFM values (28.6% and 71.4%, respectively) was achieved at 8 months, while the patient was receiving the weight maintenance dose of semaglutide (1 mg/week), which led to additional FM loss (−2.1 kg) and increases in FFM (+0.8 kg), SMM (+1.0 kg) and ASMM (+0.3 kg) with respect to the FM, FFM, SMM and ASMM values observed at 6 months (during treatment with a weekly semaglutide dose of 1.7 mg).

The abovementioned body composition changes are in line with those observed in clinical trials investigating the use of semaglutide in patients with overweight or obesity. In fact, in large clinical trials conducted in patients with overweight or obesity, once-weekly subcutaneous semaglutide therapy for 68 weeks (at a weekly dose of 2.4 mg) has been associated with a mean percent body weight loss of up to 15.8% [86,87,88]. A systematic review of randomized controlled trials and observational studies investigating the use of semaglutide in patients with overweight or obesity showed that semaglutide-mediated weight loss is predominantly attributable to FM loss, with lean mass remaining stable or decreasing by up to 40% across different studies [89]. Authors noted that decreases in lean mass were particularly evident in larger trials, although the proportion of lean mass relative to total body mass increased, thus suggesting a positive overall outcome [89]. Moreover, our results are in line with those of the SEMALEAN prospective study conducted in patients with obesity treated with once-weekly subcutaneous semaglutide (administered at a weekly dose progressively titrated up to 2.4 mg), which showed a 3-kg decline in lean mass after 7 months of treatment [90].

Importantly, GLP-1 RA therapy typically involves proportional reductions in both FM and muscle mass, reflecting expected physiological adaptations rather than pathological sarcopenia [91,92]. In fact, the proportion of lean mass relative to total body mass generally increases during incretin analog therapy (including semaglutide therapy), despite the expected lean mass reduction in absolute terms [88,90]. The incretin analog-mediated increase in lean mass relative to total body mass is typically accompanied by reductions in percent FM and visceral adipose tissue [88,90,93]. Moreover, the improvement in insulin sensitivity and skeletal muscle fat infiltration (a.k.a. myosteatosis) likely contributes to an adaptive process leading to reduced muscle volume accompanied by improved muscle quality and lower likelihood of reductions in muscle strength and function [91]. Incretin analog-mediated reductions in muscle volume also appear to be commensurate with what is expected due to disease status, aging and weight loss achieved [91]. Moreover, a recent meta-analysis showed no significant effects of GLP-1 RAs on elevated fracture risk [94]. It has also been shown that GLP-1 RAs have the potential to slow frailty progression in older adults with T2DM via mechanisms independent of cardiovascular benefits and probably involving the anti-oxidant and anti-inflammatory actions of these drugs [92,95,96].

At the end of the 8-month follow-up period, our patient also showed reductions in the absolute values (expressed in liters) of TBW, ECW, and ICW (as it has already been reported in the literature) [90,97], as well as a marginal (−0.4°) reduction in phase angle (PhA) and a 0.7-kg reduction in body cell mass (BCM), although PhA values (6.7°) remained within the reference range [98] and there was a 3.6-percentage-point increase in percent BCM. Moreover, there was a marginal reduction (−0.014) in the values of the ECW/TBW ratio, which can serve as a marker of systemic inflammation or fluid imbalance [99].

In our patient, once-weekly subcutaneous semaglutide plus low-dose metformin combination therapy (in combination with lipid-lowering therapy intensification) also resulted in notable improvements of insulin resistance, prediabetes, and hypercholesterolemia, as evidenced by normalization of HOMA-IR, QUICKI, and TyG index values, a 0.3-percentage-point reduction in HbA1c values, and the achievement of desired target serum LDL-cholesterol and non-HDL-cholesterol levels. Furthermore, semaglutide plus low-dose metformin combination therapy also resulted in the disappearance of sonographic characteristics suggestive of hepatic steatosis and in the normalization of serum GGT values, likely reflecting a meaningful improvement of MASLD, as it has already been reported [100]. Altogether, these results suggest that semaglutide and metformin may exert synergistic insulin-sensitizing effects, as previously reported in patients with T2DM and overweight/obesity [101]. Moreover, the observed improvement of insulin resistance may also be attributed to vitamin D3 supplementation, in view of the insulin-sensitizing actions of vitamin D, particularly in patients with prediabetes (as in the case of our patient) [102]. In this regard, it is worth noting that international guidelines suggest starting vitamin D supplementation (in addition to lifestyle modification) in patients with high-risk prediabetes to reduce the risk of progression to diabetes [103].

The body composition and metabolic improvements observed in our patient highlight the importance of anti-obesity pharmacotherapy, especially when there is persistent difficulty in managing excess body weight despite adherence to healthy lifestyle habits. Yet, the use of weight-loss drugs does not lessen the importance of integrated approaches to obesity management that combine pharmacotherapy with lifestyle interventions (particularly, healthy dietary patterns and structured physical activity programs) rather than replacing healthy lifestyle habits with pharmacotherapy alone [47,104,105]. Indeed, these integrated approaches enhance the therapeutic benefits and mitigate the risk of adverse effects associated with anti-obesity medications [47,104,105].

Low-dose metformin was well tolerated by the patient. Semaglutide was also well tolerated up to a weekly dose of 1.7 mg. Regarding the transient dysesthesia experienced by the patient with the highest weekly semaglutide dose (2.4 mg/week), this is a known side effect of semaglutide therapy, which appears to be more frequent at the highest doses of the drug and can resolve spontaneously over time and/or after drug discontinuation [106,107]. A similar case of semaglutide-induced dysesthesia has recently been published: a 56-year-old woman with obesity who transitioned from daily liraglutide therapy (at a dose of 3 mg/day) to once-weekly semaglutide (at a dose of 2.4 mg/week) started to complain of dysesthesia, reporting a sunburn-like skin sensation within a few weeks of initiating semaglutide therapy [106]. However, dysesthesia persisted for approximately 6 weeks before resolving spontaneously [106]. Notably, dysesthesia has been reported as a potential adverse effect of subcutaneous semaglutide in the STEP UP and STEP UP T2D trials, although it was dose-dependent and occurred at a higher frequency with the 7.2 mg weekly semaglutide dose than with the 2.4 mg weekly semaglutide dose or placebo [108,109].

Finally, we acknowledge that the results of a single case report cannot be generalized to broader patient populations. Therefore, future case series and prospective cohort studies are certainly required to confirm our observations in patients with various numerical and structural X chromosome abnormalities (including partial Xq deletions), comorbid overweight/obesity and related metabolic disorders.

## 4. Conclusions

Once-weekly subcutaneous semaglutide plus low-dose metformin combination therapy (prescribed as an adjunct to lifestyle intervention) was well tolerated and effective for the treatment of obesity and prediabetes in a woman with a partial deletion of the X chromosome long arm. This case report highlights the importance of a timely approach to the management of complex cases of obesity, integrating early pharmacological treatment with lifestyle counseling and metabolic rehabilitation. However, large case series and prospective cohort studies are warranted to better investigate the safety and efficacy profile of semaglutide (alone or in combination with metformin) in patients with different numerical and structural X chromosome abnormalities (including partial Xq deletions), comorbid overweight/obesity and related metabolic disorders.

## Figures and Tables

**Table 1:** Patient’s anthropometric parameters and body circumferences at baseline (before initiation of semaglutide plus low-dose metformin combination therapy) and during the follow-up period.

Parameter	Baseline	2 Months	4 Months	6 Months	8 Months	Change from Baseline
Body height (cm)	144.0	144.0	144.0	144.0	144.0	Unchanged
Body weight (kg)	65.9	62.2	56.6	53.4	52.1	−13.8 (%TBWL: 20.95%)
BMI (kg/m^2^)	31.8	30.0	27.3	25.8	25.1	−6.7
Bust circumference (cm)	109.9	107.3	101.4	97.9	98.9	−11.0
WC (cm)	101.2	97.6	93.1	89.9	87.1	−14.1
Hip circumference (cm)	106.3	103.5	98.5	96.5	95.0	−11.3
WHR	0.95	0.94	0.94	0.93	0.91	−0.04
WHtR	0.70	0.67	0.64	0.62	0.60	−0.10
Left arm circumference (cm)	31.0	30.4	29.7	27.7	28.3	−2.7
Right arm circumference (cm)	32.0	31.2	28.7	29.4	28.4	−3.6
Left forearm circumference (cm)	27.4	26.6	27.4	25.3	26.0	−1.4
Right forearm circumference (cm)	26.3	25.6	24.6	24.3	24.0	−2.3
Left thigh circumference (cm)	57.8	55.8	54.0	51.1	50.4	−7.4
Right thigh circumference (cm)	59.1	57.3	54.4	53.2	52.5	−6.6
Left calf circumference (cm)	43.9	43.2	43.6	40.0	39.3	−4.6
Right calf circumference (cm)	43.1	42.0	40.8	39.4	38.8	−4.3

Body circumferences were assessed by three-dimensional (3D) body scanning technology, using the Fit3D ProScanner (Fit3D ProScanner v5.0; Fit3D, Inc.; San Mateo, CA, USA). Abbreviations: %TBWL, percent total body weight loss; BMI, body mass index; WC, waist circumference; WHR, waist-to-hip ratio; WHtR, waist-to-height ratio.

**Table 2:** Patient’s bioelectrical impedance analysis (BIA) parameters at baseline (before initiation of semaglutide plus low-dose metformin combination therapy) and during the follow-up period.

Parameter	Baseline	2 Months	4 Months	6 Months	8 Months	Change from Baseline
Body height (cm)	144.0	144.0	144.0	144.0	144.0	Unchanged
Body weight (kg)	65.9	62.2	56.6	53.4	52.1	−13.8 (%TBWL: 20.95%)
BMI (kg/m^2^)	31.8	30.0	27.3	25.8	25.1	−6.7
Rz (Ω)	381.3	436.6	439.1	440.3	417.8	+36.5
Xc (Ω)	47.5	54.9	50.7	50.8	49.4	+1.9
PhA (°)	7.1	7.2	6.6	6.6	6.7	−0.4
TBW (L)	35.5	32.0	31.2	30.8	31.8	−3.7
TBW (%)	53.9%	51.4%	55.1%	57.7%	61.0%	+7.1%
ECW (L)	16.9	14.9	14.6	14.3	14.7	−2.2
ECW (%)	47.6%	46.6%	46.8%	46.4%	46.2%	−1.4%
ICW (L)	18.6	17.1	16.6	16.5	17.1	−1.5
ICW (%)	52.4%	53.4%	53.2%	53.6%	53.8%	+1.4%
ECW/TBW ratio	0.476	0.465	0.467	0.464	0.462	−0.014
FFM (kg)	40.7	38.8	37.0	36.4	37.2	−3.5
FFM (%)	61.8%	62.4%	65.4%	68.2%	71.4%	+9.6%
FFMI (kg/m^2^)	19.6	18.7	17.8	17.5	17.9	−1.7
FM (kg)	25.2	23.4	19.6	17.0	14.9	−10.3
FM (%)	38.2%	37.6%	34.6%	31.8%	28.6%	−9.6%
FMI (kg/m^2^)	12.1	11.3	9.4	8.2	7.2	−4.9
FM/FFM ratio	0.61	0.60	0.52	0.46	0.40	−0.21
BCM (kg)	23.8	22.4	21.8	22.0	23.1	−0.7
BCM (%)	58.5%	57.7%	58.9%	60.4%	62.1%	+3.6%
BCMI (kg/m^2^)	11.4	10.8	10.5	10.6	11.1	−0.3
SMM * (kg)	22.3	19.5	19.4	19.3	20.3	−2.0
SMM * (%)	33.8%	31.4%	34.3%	36.1%	39.0%	+5.2%
ASMM (kg)	17.7	16.2	15.4	15.1	15.4	−2.3
ASMM (%)	26.9%	26.0%	27.2%	28.3%	29.6%	+2.7%
BMR (kcal/24 h)	1541.2	1469.6	1439.0	1449.2	1505.4	−35.8

Body composition was assessed by bioelectrical impedance analysis (BIA), using the BIA 101 BIVA^®^ PRO (Akern S.r.l., Pontassieve, Florence, Italy). BMI was calculated using the following formula: body weight (kg)/height (m^2^). BCMI was calculated using the following formula: BCM (kg)/height (m^2^). FFMI was calculated using the following formula: FFM (kg)/height (m^2^). FMI was calculated using the following formula: FM (kg)/height (m^2^). ECW/TBW was calculated by dividing ECW (expressed in liters) by TBW (expressed in liters), while the FM/FFM ratio was calculated by dividing FM (expressed in kg) by FFM (expressed in kg). * SMM was estimated using the Janssen’s equation, as previously described (Janssen et al. 2000 [32]). Abbreviations: %TBWL, percent total body weight loss; ASMM, appendicular skeletal muscle mass; BCM, body cell mass; BCMI, body cell mass index; BMI, body mass index; BMR, basal metabolic rate; ECW, extracellular water; FFM, fat-free mass; FFMI, fat-free mass index; FM, fat mass; FMI, fat mass index; ICW, intracellular water; kg, kilograms; L, liters; PhA, phase angle; Rz, resistance; SMM, skeletal muscle mass; TBW, total body water; Xc, reactance; Ω, Ohms.

**Table 3:** Dosing regimen used for semaglutide therapy during the follow-up period.

Month	Semaglutide Dose
Month 1 (weeks 1 through 4)	0.25 mg/week
Month 2 (weeks 5 through 8)	0.5 mg/week
Month 3 (weeks 9 through 12)	1 mg/week
Month 4 (weeks 13 through 16)	1.7 mg/week
Month 5 (weeks 17 through 20)	2.4 mg/week
Month 6 (weeks 21 through 24)	1.7 mg/week
Months 7 and 8 (weeks 25 through 32)	1 mg/week

## Data Availability

All data analyzed in this study are included in the present article and in its Supplementary Materials.

## Supplementary Materials

The following supporting information can be downloaded at: https://www.mdpi.com/article/10.3390/reports9010075/s1; Supplementary Table S1: Results of the laboratory tests at baseline (before initiation of semaglutide plus low-dose metformin combination therapy) and during the follow-up period.
