# Supplementary material for: Semaglutide Plus Low-Dose Metformin Combination Therapy for the Treatment of Obesity and Prediabetes in a Woman with Partial Deletion of the X Chromosome Long Arm
Source: Reports (MDPI). 2026 Feb 28;9(1):75. doi: 10.3390/reports9010075 (PMC13030616; doi:10.3390/reports9010075)
Supplement: Supplementary file 1 [file reports-09-00075-s001.zip › reports-4147257-supplementary.pdf]

Supplementary Table S1. Results of the laboratory tests at baseline (before initiation of semaglutide plus low-dose metformin combination therapy) and during the follow-up period.

Blood parameter	Baseline	5 months	Change from baseline
FPG (mg/dL) [RR: 70-99 mg/dL]	96.0	87.0	-9.0
Fasting insulinemia (μIU/mL) [RR: 2.5-24.9 μIU/mL]	14.65	7.5	-7.15
HOMA-IR [RR: <2.5]	3.5	1.6	-1.9
QUICKI [RR: >0.33]	0.317	0.355	+0.038
Fasting serum C-peptide (ng/mL) [RR: 0.78-2.7 ng/mL]	n/a	1.93	Not applicable
HbA1c (%) [RR: <5.7%]	6.0	5.7	-0.3
HbA1c (mmol/mol) [RR: <39 mmol/mol]	42.0	39.0	-3.0
WBC count (x10^3/μL) [RR: 4.00-10.00x10^3/μL]	5.89	8.52	+2.63
Absolute neutrophil count (x10^3/μL) [RR: 1.80-7.00x10^3/μL]	2.88	4.22	+1.34
Percentage of neutrophils (%) [RR: 40.0-75.0%]	48.9	49.5	+0.6
Absolute lymphocyte count (x10^3/μL) [RR: 1.00-4.80x10^3/μL]	2.62	3.71	+1.09
Percentage of lymphocytes (%) [RR: 20.0-45.0%]	44.5	43.6	-0.9
Absolute monocyte count (x10^3/μL) [RR: 0.20-0.80x10^3/μL]	0.20	0.39	+0.19
Percentage of monocytes (%) [RR: 2.0-8.0%]	3.4	4.6	+1.2
Absolute eosinophil count (x10^3/μL) [RR: 0.03-0.50x10^3/μL]	0.17	0.14	-0.03
Percentage of eosinophils (%) [RR: 1.0-4.9%]	2.9	1.7	-1.2
Absolute basophil count (x10^3/μL) [RR: 0.00-0.30x10^3/μL]	0.02	0.06	+0.04

Percentage of basophils (%) [RR: 0.1-1.0%]	0.3	0.7	+0.4
RBC count (x10^6/µL) [RR: 4.2-5.4x10^6/µL]	4.22	4.52	+0.30
Hemoglobin (g/dL) [RR: 12.3-15.3 g/dL]	12.4	13.2	+0.8
Hematocrit (%) [RR: 36.0-44.0%]	37.9	40.7	+2.8
MCV (fL) [RR: 80.0-100.0 fL]	89.8	90.1	+0.3
MCH (pg/cell) [RR: 27.0-33.0 pg/cell]	29.4	29.3	-0.1
MCHC (g/dL) [RR: 32.0-36.0 g/dL]	32.7	32.5	-0.2
RDW (%) [RR: 12.0-15.0%]	12.9	13.5	+0.6
Platelet count (x10^3/µL) [RR: 150.0-450.0x10^3/µL]	266.0	316.0	+50.0
ESR (mm/h) [RR: <30 mm/h]	15.0	10.0	-5.0
CRP (mg/dL) [RR: 0.00-0.50 mg/dL]	n/a	<0.04	Not applicable
Total cholesterol (mg/dL) [RR: <200 mg/dL]	197.0	137.0	-60.0
LDL cholesterol (mg/dL)* [RR: <100 mg/dL]	102.2	50.0	-52.2
HDL cholesterol (mg/dL) [RR: 50.0-80.0 mg/dL]	66.0	62.0	-4.0
Non-HDL cholesterol (mg/dL) [RR: <130 mg/dL]	131.0	75.0	-56.0
Total cholesterol/HDL cholesterol ratio [RR: <4.0]	2.98	2.20	-0.78
Triglycerides (mg/dL) [RR: <150 mg/dL]	144.0	92.0	-52.0
TyG index [RR: <4.5]	4.77	4.49	-0.28
AST (U/L) [RR: 8.00-40.00 U/L]	24.0	28.0	+4.0
ALT (U/L) [RR: 7.00-45.00 U/L]	23.0	31.0	+8.0
GGT (U/L) [RR: 2.0-35.00 U/L]	39.0	22.0	-17.0
ALP (U/L) [RR: 46.0-122.0 U/L]	n/a	54.0	Not applicable

CPK (U/L) [RR: 30.0-170.0 U/L]	189.0	79.0	-110.0
Urea (mg/dL) [RR: 21.00-43.0 mg/dL]	21.0	25.0	+4.0
Serum creatinine (mg/dL) [RR: 0.59-1.04 mg/dL]	0.80	0.73	-0.07
eGFR (mL/min/1.73 m^2)** [RR: ≥90 mL/min/1.73 m^2]	82	91	+9.0
Bilirubin (total with reflex; mg/dL) [RR: 0.20-1.20 mg/dL]	0.47	0.76	+0.29
Homocysteine (µmol/L) [RR: 5.0-14.9 µmol/L]	n/a	9.0	Not applicable
TSH (mIU/L) [RR: 0.40-4.94 mIU/L]	3.02	2.74	-0.28
FT3 (pg/mL) [RR: 2.3-4.1 pg/mL]	3.59	n/a	Not applicable
FT4 (ng/dL) [RR: 0.9-1.8 ng/dL]	0.98	n/a	Not applicable
Anti-thyroid peroxidase (TPO) antibodies (IU/mL) [RR: <5.6 IU/mL]	0.10	n/a	Not applicable
Anti-thyroglobulin antibodies (IU/mL) [RR: <4 IU/mL]	0.10	n/a	Not applicable
Calcitonin (pg/mL) [RR: <5.0 pg/mL]	0.88	n/a	Not applicable
PTH (pg/mL) [RR: 15.0-65.0 pg/mL]	n/a	57.6	Not applicable
Total serum protein (g/L) [RR: 60.0-80.0 g/L]	72.0	75.0	+3.0
Albumin (g/L) [RR: 35.0-55.0 g/L]	43.2	45.7	+2.5
Alpha-1 globulins (g/L) [RR: 1.0-3.0 g/L]	4.6	2.6	-2.0
Alpha-2 globulins (g/L) [RR: 6.0-10.0 g/L]	6.4	7.0	+0.6
Beta-1 globulins (g/L) [RR: 4.0-6.0 g/L]	4.6	4.6	Unchanged
Beta-2 globulins (g/L) [RR: 2.0-4.0 g/L]	3.5	3.8	+0.3
Gamma globulins (g/L)	9.7	11.3	+1.6

[RR: 7.00-16.00 g/L]			
A/G ratio [RR: 1.1-2.5]	1.50	1.56	+0.06
25(OH)-vitamin D (ng/mL) [RR (vitamin D sufficiency): ≥30.0 ng/mL]	12.14	39.0	+26.86
Vitamin B12 (pg/mL) [RR: 200.0-950.0 pg/mL]	n/a	299.0	Not applicable
Folate (ng/mL) [RR: 3.0-20.5 ng/mL]	n/a	11.3	Not applicable
Sodium (mmol/L) [RR: 135.0-145.0 mmol/L]	n/a	140.0	Not applicable
Potassium (mmol/L) [RR: 3.5-5.0 mmol/L]	n/a	4.4	Not applicable
Total serum calcium (mg/dL) [RR: 8.5-10.2 mg/dL]	9.48	9.50	+0.02
Phosphate (mg/dL) [RR: 2.5-4.5 mg/dL]	n/a	4.4	Not applicable
Magnesium (mg/dL) [RR: 1.7-2.4 mg/dL]	n/a	2.2	Not applicable
Uric acid (mg/dL) [RR: 2.5-5.9 mg/dL]	n/a	3.0	Not applicable
Iron (µg/dL) [RR: 50.0-170.0 µg/dL]	n/a	87.0	Not applicable
Transferrin (mg/dL) [RR: 215-360 mg/dL]	n/a	300.0	Not applicable
Ferritin (ng/mL) [RR: 15-205 ng/mL]	n/a	146.0	Not applicable
Amylase (U/L) [RR: 28.0-100.0 U/L]	n/a	78.0	Not applicable
Lipase (U/L) [RR: 14-72 U/L]	n/a	58.0	Not applicable
HBsAg [chemiluminescence immunoassay; RR: <0.005 IU/mL]	n/a	0.0011	Not applicable
HCV-Ab [chemiluminescence immunoassay; RR: Non-reactive]	n/a	Non-reactive	Not applicable

Urinalysis	Baseline	5 months	Change from baseline
Urine pH RR: 5.5-7.5; urine specific gravity RR: 1005-1030; uACR RR: <30 mg/g.			
	Urine color: pale yellow; urine pH 6.0; urine specific gravity 1025; uACR: n/a; glucose, ketones or nitrites were not detected in the urine; absence of proteinuria; negative leukocyte esterase urine test; absence of bilirubinuria, bacteriuria, leukocyturia and hematuria; absence of other abnormal urinalysis findings.	Urine color: pale yellow; urine pH 5.5; urine specific gravity 1014; uACR 15 mg/g; glucose, ketones or nitrites were not detected in the urine; absence of proteinuria; negative leukocyte esterase urine test; absence of bilirubinuria, bacteriuria, leukocyturia and hematuria; absence of other abnormal urinalysis findings.	Comparable (normal) urinalysis results

Laboratory tests were performed after a 10-hour overnight fast. *At baseline, LDL cholesterol was calculated using the Friedewald equation (ref. 41; Friedewald et al. 1972); at 5 months from baseline, LDL cholesterol level measured by direct LDL-C assay (homogenous enzyme assay) was 50.0 mg/dL, whereas LDL cholesterol level measured using the Friedewald equation was 56.6 mg/dL. **eGFR was calculated using the 2021 Chronic Kidney Disease Epidemiology Collaboration (CKD-EPI) equation (ref. 42; Lu et al. 2023) Abbreviations: 25(OH)-vitamin D, 25-hydroxyvitamin D; A/G ratio, Albumin/globulin ratio; ALP, Alkaline phosphatase; ALT, Alanine aminotransferase; AST, Aspartate aminotransferase; CPK, Creatine phosphokinase; CRP, C-reactive protein; eGFR, Estimated glomerular filtration rate; ESR, Erythrocyte sedimentation rate; FPG, Fasting plasma glucose; FT3, Free triiodothyronine; FT4, Free thyroxine; GGT, Gamma-glutamyl transferase; HbA1c, Glycated hemoglobin; HBsAg, Hepatitis B surface antigen; HCV-Ab, Hepatitis C virus antibodies; HDL, High-density lipoprotein; HOMA-IR, Homeostatic model assessment for insulin resistance; LDL, Low-density lipoprotein; MCH, Mean corpuscular hemoglobin; MCHC, Mean corpuscular hemoglobin concentration; MCV, Mean corpuscular volume; n/a, not available; PTH, Parathyroid hormone; QUICKI, Quantitative insulin sensitivity check index; RBC, Red blood cell; RDW, Red Cell Distribution Width; RR, Reference range; TSH, Thyroid-stimulating hormone; TyG index, Triglyceride-glucose index; uACR, Urine albumin-to-creatinine ratio; WBC, White blood cell.
